# Dynamical modeling of microRNA action on the protein translation process

**DOI:** 10.1186/1752-0509-4-13

**Published:** 2010-02-24

**Authors:** Andrei Zinovyev, Nadya Morozova, Nora Nonne, Emmanuel Barillot, Annick Harel-Bellan, Alexander N Gorban

**Affiliations:** 1 Institut Curie, Bioinformatics and Computational Systems Biology Of Cancer, Paris, France; 2 INSERM, U900, Paris, F-75248 France; 3 Mines ParisTech, Centre for Computational Biology, Fontainebleau, F-77300 France; 4 CNRS FRE 2944, Institut André Lwoff, Villejuif, France; 5 University of Leicester, Center for Mathematical Modeling, Leicester, UK; 6 Institute of Computational Modeling SB RAS, Department of nonequilibrium systems, Krasnoyarsk, Russia

## Abstract

**Background:**

Protein translation is a multistep process which can be represented as a cascade of biochemical reactions (initiation, ribosome assembly, elongation, etc.), the rate of which can be regulated by small non-coding microRNAs through multiple mechanisms. It remains unclear what mechanisms of microRNA action are the most dominant: moreover, many experimental reports deliver controversial messages on what is the concrete mechanism actually observed in the experiment. Nissan and Parker have recently demonstrated that it might be impossible to distinguish alternative biological hypotheses using the steady state data on the rate of protein synthesis. For their analysis they used two simple kinetic models of protein translation.

**Results:**

In contrary to the study by Nissan and Parker, we show that dynamical data allow discriminating some of the mechanisms of microRNA action. We demonstrate this using the same models as developed by Nissan and Parker for the sake of comparison but the methods developed (asymptotology of biochemical networks) can be used for other models. We formulate a hypothesis that the effect of microRNA action is measurable and observable only if it affects the dominant system (generalization of the limiting step notion for complex networks) of the protein translation machinery. The dominant system can vary in different experimental conditions that can partially explain the existing controversy of some of the experimental data.

**Conclusions:**

Our analysis of the transient protein translation dynamics shows that it gives enough information to verify or reject a hypothesis about a particular molecular mechanism of microRNA action on protein translation. For multiscale systems only that action of microRNA is distinguishable which affects the parameters of dominant system (critical parameters), or changes the dominant system itself. Dominant systems generalize and further develop the old and very popular idea of limiting step. Algorithms for identifying dominant systems in multiscale kinetic models are straightforward but not trivial and depend only on the ordering of the model parameters but not on their concrete values. Asymptotic approach to kinetic models allows putting in order diverse experimental observations in complex situations when many alternative hypotheses co-exist.

## Background

MicroRNAs (miRNAs) are currently considered as key regulators of a wide variety of biological pathways, including development, differentiation and oncogenesis. Recently, remarkable progress was made in understanding of microRNA biogenesis, functions and mechanisms of action. Mature microRNAs are incorporated into the RISC effector complex, which includes as a key component an Argonaute protein. MicroRNAs affect gene expression by guiding the RISC complex toward specific target mRNAs. The exact mechanism of this inhibition is still a matter of debate. In the past few years, several mechanisms have been reported, and some of the reports contradict to each other (for review, see [1-3]). The inhibition mechanisms include, in particular, the inhibition of translation initiation (acting at the level of cap-40S or 40S-AUG-60S association steps), the inhibition of translation elongation and the premature translation termination. MicroRNA-mediated mRNA decay and sequestration of target mRNAs in P-bodies have been also proposed. Moreover, some microRNAs mediate target mRNA cleavage [4], chromatin reorganization followed by transcriptional repression or activation [5,6], or translational activation [7,8].

**Figure 1:**
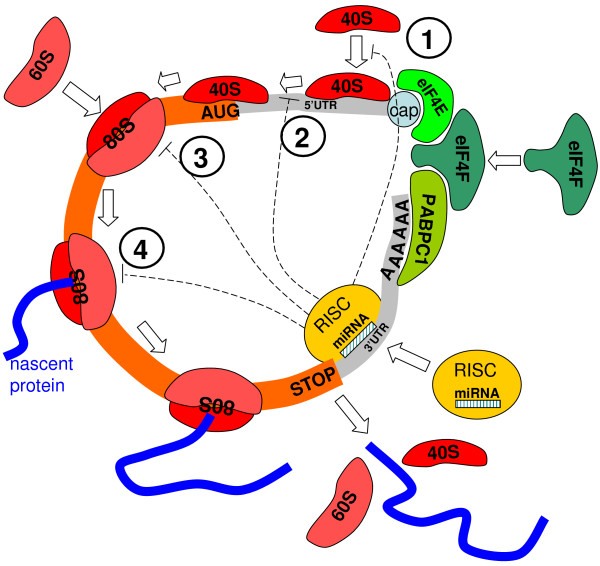
**Interaction of microRNA with protein translation process**. Four mechanisms of translation repression which are considered in the mathematical modeling are indicated: 1) on the initiation process, preventing assembling of the initiation complex; 2) on a late initiation step, such as searching for the start codon; 3) on the ribosome assembly; 4) on the translation process. There exist other mechanisms of microRNA action on protein translation (transcriptional, transport to P-bodies, ribosome drop-off, co-translational protein degradation and others) that are not considered in this paper. Here 40S and 60S are light and heavy components of the ribosome, 80S is the assembled ribosome bound to mRNA, eIF4F is an translation initiation factor, PABC1 is the Poly-A binding protein, "cap" is the mRNA cap structure needed for mRNA circularization, RISC is the RNA-induced silencing complex.

The most frequently reported, but also much debated, is the mechanism of gene repression by microRNAs which occurs at the level of mRNA translation. At this level, several mode of actions have been suggested (see Fig. 1). Historically, the first proposed mechanism was the inhibition of translation elongation. The major argument supporting this hypothesis was the observation that the inhibited mRNA remained associated with the polysomal fraction (in which mRNAs are associated with polysomes). This observation was reproduced in different systems [9-13]. The idea of a post-initiation mechanism was further supported by the observation that some mRNAs can be repressed by a microRNA even when their translation is cap-independent (mRNAs with an IRES or A-capped) [11,14-16]. Although it was initially proposed that the ribosomes were somehow "frozen" on the mRNA, it is important to note that it is difficult to discriminate experimentally between different potential post-initiation mechanisms, such as elongation inhibition, premature ribosome dissociation ("ribosome drop-off") or normal elongation but with nascent polypeptide degradation. The last proposition (this mechanism can occur in conjunction with the two others) is supported by the fact that the mRNA-polysomal association is puromycin-sensitive, indicating that it depends on a peptidyl-transferase activity [13,17]. However, no nascent peptide has ever been experimentally demonstrated; thus its degradation would occur extremely rapidly after the synthesis [10,11,18]. The premature ribosome dissociation is supported by the decreased read-through of inhibited mRNA [11]. The ribosomal drop-off and/or ribosomal "slowing" are supported by the slight decrease in the number of associated ribosomes observed in some studies [10,13].

Concurrently, several reports have been published indicating an action of microRNAs at the level of initiation. An increasing number of papers reports that microRNA-targeted mRNAs shift towards the light fractions in polysomal profiles [18-20]. This shows a decrease of mature translating ribosomes, suggesting that microRNAs act at the initiation step. Moreover, several reports show that microRNA-mediated inhibition is relieved when translation is driven by a cap-independent mechanism such as IRES-mRNA or A-capped-mRNA [18,20,21]. This observation was confirmed in several in-vitro studies [22-25]. In particular, in one of those, an excess of eIF4F could relieve the inhibition, and the inhibition led to the decreased 80S in the polysomal gradient [22].

Most of the data indicating a shift towards the light polysomal fraction or the requirement for a cap-dependent translation are often interpreted in favour of involvement of microRNAs at early steps of translation, i.e., cap binding and 40S recruitment. However, some of them are also compatible with a block at the level of 60S subunit joining. This hypothesis is also supported by in-vitro experiments showing a lower amount of 60S relative to 40S on inhibited mRNAs. Moreover, toe-printing experiments show that 40S is positioned on the AUG [26]. Independently, it was shown that eIF6, an inhibitor of 60S joining, is required for microRNA action [27], but this was in contradiction with other studies [2].

*Thus, the data on the exact step of translational inhibition are clearly contradictory*. Taking also into account the data about mRNA degradation and P-bodies localization, it is difficult to draw a clear picture of the situation, and the exact mechanism by which microRNA represses mRNA expression is highly controversial, not mentioning the interrelations between the different mechanisms and their possible concomitant action. Several attempts to integrate the different hypotheses have been made [1-3,28-30]. For example, it was proposed that one mechanism could act as a "primary" effect, and the other as a "secondary" mechanism, either used to reinforce the inhibition or as a back-up mechanism. In others, the different mechanisms could all coexist, but occur differentially depending on some yet unidentified characteristics. For example, it has been observed than the same mRNA targeted by the same microRNA can be regulated either at the initiation or the elongation step depending on the mRNA promoter and thus on the mRNA nuclear history [31]. It was also proposed that technical (experimental) problems, including the variety of experimental systems used, may also account for these discrepancies [1-3]. However, this possibility does not seem to be sufficient to provide a simple and convincing explanation to the reported discrepancies.

A possible solution to exploit the experimental observations and to provide a rational and straightforward data interpretation is the use of mathematical models for microRNA action on protein translation. For many years, the process of protein synthesis is a subject of mathematical modeling with use of various approaches from chemical kinetics and theoretical physics. Many of the models created consider several stages of translation, however, most of them concentrate on the elongation and termination processes. In [32-34], a non-equilibrium statistical physics description of protein synthesis was proposed. Models taking into account gene sequence were developed in [33,35-37]. These models can predict the probability of that a ribosome will completely terminate a transcript, spatio-temporal organization of ribosomes in polysome, dependence of the protein synthesis rate on various factors, such as presence of slow synonymous codons in the gene sequence [33,37] and the frequency of non-sense errors [35]. Several models of the effect of microRNA on protein translation were proposed. Thus, in [38] the authors tried to determine which inhibition mechanism (via translation repression or transcript degradation) is the most abundant in mammalian cells using Bayesian modeling and microarray data. Quantitative features of sRNA-mediated gene regulation were considered in [39]. A simple kinetic model of microRNA-mediated mRNA degradation was proposed in [40] and compared to a temporal microarray dataset.

In this paper we will analyze two simple models of microRNA action on protein translation developed recently by Nissan and Parker [41]. They studied the microRNA-dependent steady states rates of protein synthesis [41] and provided a critical analysis for the experiments with alternative mRNA cap structures and IRES elements [22,23,25]. This analysis led to a possible explanation of the conflicting results. The authors suggested that the relief of translational repression upon replacement of the cap structure can be explained if microRNA is acting on a step which is not rate-limiting in the modified system, in which case, the effect of microRNA can simply not be observed. It was claimed that it is impossible to discriminate between two alternative interpretations of the biological experiments with cap structure replacement, using sole monitoring of the steady state level of protein synthesis [41].

Two remarks can be made in this regard. Firstly, in practice not only the steady state level of protein can be observed but also other dynamical characteristics, such as the *relaxation time*, i.e. the time needed to achieve the steady state rate after a perturbation (such as restarting the translation process). We argue that having these measurements in hands, *one can distinguish between two alternative interpretations*. In this paper we provide such a method from the same models as constructed by Nissan and Parker, for comparison purposes. However, the method applied can be easily generalized for other models.

Secondly, even in the simple non-linear model of protein translation, taking into account the recycling of ribosomal components, it is difficult to define what is the rate limiting step. It is known from the theory of asymptotology of biochemical networks [42] that even in complex linear systems the "rate limiting place" notion is not trivial and cannot be reduced to a single reaction step. Moreover, in non-linear systems the "rate limiting place" can change with time and depend on the initial conditions. Hence, conclusions of [41] should be re-considered for the non-linear model, made more precise and general. The notion of rate limiting step should be replaced by the notion of *dominant system*.

In this paper we perform careful analysis of the Nissan and Parker's models and provide their approximate analytical solutions, which allows us to generalize the conclusions of [41] and make new checkable predictions on the identifiability of active mechanism of microRNA-dependent protein translation inhibition.

The paper is organized in the following way. The Methods contain introduction, all necessary definitions and basic results of the asymptotology of biochemical reaction networks (*quasiequilibrium, quasi steady-state, limiting step and dominant system asymptotics*), used further in the Results. The Methods section is deliberately made rather detailed to make the reading self-sufficient. These details are necessary for reproducing the analytical calculations but not for understanding the interpretation of the modeling results. When the most important notions are introduced (such as dominant system, critical parameters), they are emphasized in bold. The Results section starts with listing model assumptions, followed by deriving semi-analytical solutions of Nissan and Parker's model and interpretation of the analysis results and prediction formulations. For those readers who are interested only in the applied aspect of this work, it is possible to skip the details of deriving the analytical solutions and start reading from the "Model assumptions" section, look at the definition of the model parameters and variables and continue with 'the 'Effect of microRNA on the translation dynamics" section.

## Results

### Model assumptions

We consider two models of action of microRNA on protein translation process proposed in [41]: the simplest linear model, and the non-linear model which explicitly takes into account recycling of ribosomal subunits and initiation factors.

Both models, of course, are significant simplifications of biological reality. Firstly, only a limited subset of all possible mechanisms of microRNA action on the translation process is considered (see Fig. 1). Secondly, all processes of synthesis and degradation of mRNA and microRNA are deliberately neglected. Thirdly, interaction of microRNA and mRNA is simplified: it is supposed that when microRNA is added to the experimental system then only mRNA with bound microRNAs are present (this also assumes that the concentration of microRNA is abundant with respect to mRNA). Concentrations of microRNA and mRNA are supposed to be constant. Interaction of only one type of microRNA and one type of mRNA is considered (not a mix of several microRNAs). The process of initiation is greatly simplified: all initiation factors are represented by only one molecule which is marked as eIF4F.

Finally, the classical chemical kinetics approach is applied, based on solutions of ordinary differential equations, which supposes sufficient and well-stirred amount of both microRNAs and mRNAs. Another assumption in the modeling is the mass action law assumed for the reaction kinetic rates.

It is important to underline the interpretation of certain chemical species considered in the system. The ribosomal subunits and the initiation factors in the model exist in free and bound forms, moreover, the ribosomal subunits can be bound to several regions of mRNA (the initiation site, the start codon, the coding part). Importantly, several copies of fully assembled ribosome can be bound to one mRNA. To model this situation, we have to introduce the following quantification rule for chemical species: amount of "ribosome bound to mRNA" means the total number of ribosomes translating proteins, which is not equal to the number of mRNAs with ribosome sitting on them, since one mRNA can hold several translating ribosomes (polyribosome). In this view, mRNAs act as *places *or *catalyzers*, where translation takes place, whereas mRNA itself formally is not consumed in the process of translation, but, of course, can be degraded or synthesized (which is, however, not considered in the models described further).

### The simplest linear protein translation model

**Figure 2:**
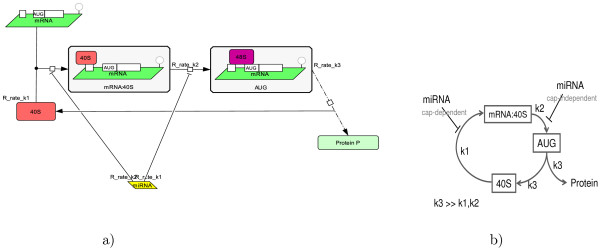
**The simplest model of microRNA action on the protein translation**. The simplest model of microRNA action on the protein translation, represented with use of Systems Biology Graphical Notation (a) and schematically with the condition on the constants (b). The two mechanisms of microRNA action (cap-dependent and cap-independent) are depicted.

The simplest representation of the translation process has the form of a circular cascade of reactions [41] (see Fig. 2).

The list of chemical species in the model is the following:

1. 40S, free small ribosomal subunit.

2. mRNA:40S, small ribosomal subunit bound to the initiation site.

3. AUG, small ribosomal subunit bound to the start codon.

The catalytic cycle is formed by the following reactions:

1. 40S → mRNA:40S, Initiation complex assembly (rate *k*_1_).

2. mRNA:40S → AUG, Some late and cap-independent initiation steps, such as scanning the 5'UTR for the start AUG codon recognition (rate *k*_2_) and 60S ribosomal unit joining.

3. AUG → 40S, combined processes of protein elongation and termination, which leads to production of the protein (rate *k*_3_), and fall off of the ribosome from mRNA.

The model is described by the following system of equations [41]:(1)

where *Prsynth*(*t*) is the rate of protein synthesis.

Following [41], let us assume that *k*_3 _>>*k*_1_, *k*_2_. This choice was justified by the following statement: "...The subunit joining and protein production rate (*k*_3_) is faster than *k*_1 _and *k*_2 _since mRNA:40S complexes bound to the AUG without the 60S subunit are generally not observed in translation initiation unless this step is stalled by experimental methods, and elongation is generally thought to not be rate limiting in protein synthesis..." [41].

Under this condition, the equations (1) have the following approximate solution (which becomes the more exact the smaller the  ratio):(2)

for the initial condition(4)

From the solution (2) it follows that the dynamics of the system evolves on two time scales: 1) fast elongation dynamics on the time scale ≈ 1/*k*_3_; and 2) relatively slow translation initiation dynamics with the relaxation time . The protein synthesis rate formula (3) does not include the *k*_3 _rate, since it is neglected with respect to *k*_1_, *k*_2 _values. From (3) we can extract the formula for the protein synthesis steady-state rate *Prsynth *(multiplier before the parentheses) and the relaxation time *t*_*rel *_for it (inverse of the exponent power):(5)

Now let us consider two experimental situations: 1) the rates of the two translation initiation steps are comparable *k*_1 _≈ *k*_2_; 2) the cap-dependent rate *k*_1 _is limiting: *k*_1 _<<*k*_2_. Accordingly to [41], the second situation can correspond to modified mRNA with an alternative cap-structure, which is much less efficient for the assembly of the initiation factors, 40S ribosomal subunit and polyA binding proteins.

**Figure 3:**
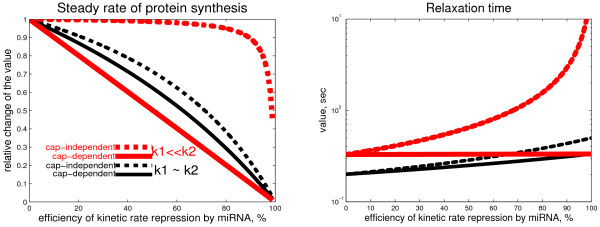
**Predicted change in the steady-state rate of protein synthesis and its relaxation time**. Graphs illustrating the predicted change in the steady-state rate of protein synthesis (left), and its relaxation time, i.e., the time needed to recover from a perturbation to the steady state value (right). Four curves are presented. The black ones are for the wild-type cap structure, which is modeled by *k*_1 _= *k*_2_. The red ones are for the modified structure, when *k*_1 _<<*k*_2_. The main conclusion from the left graph is that if microRNA acts on a late initiation step, diminishing *k*_2_, then its effect is not measurable unless *k*_2 _is very strongly suppressed (as reported in [41]). The main conclusion from the right graph is that the effect of microRNA can be measurable in this case if one looks at dynamical features such the relaxation time.

For these two experimental systems (let us call them "wild-type" and "modified" correspondingly), let us study the effect of microRNA action. We will model the microRNA action by diminishing the value of a kinetic rate coefficient for the reaction representing the step on which the microRNA is acting. Let us assume that there are two alternative mechanisms: 1) microRNA acts in a cap-dependent manner (thus, reducing the *k*_1 _constant) and 2) microRNA acts in a cap-independent manner, for example, through interfering with 60S subunit joining (thus, reducing the *k*_2 _constant). The dependence of the steady rate of protein synthesis  and the relaxation time  on the efficiency of the microRNA action (i.e., how much it is capable to diminish a rate coefficient) is shown on Fig. 3.

Interestingly, experiments with cap structure replacement were made and the effect of microRNA action on the translation was measured [22,23]. No change in the protein rate synthesis after applying microRNA was observed. From this it was concluded that microRNA in this system should act through a cap-dependent mechanism (i.e., the normal "wild-type" cap is required for microRNA recruitment). It was argued that this could be a misinterpretation [41] since in the "modified" system, cap-dependent translation initiation is a rate limiting process (*k*_1 _<<*k*_2_), hence, even if microRNA acts in the cap-independent manner (inhibiting *k*_2_), it will have no effect on the final steady state protein synthesis rate. This was confirmed this by the graph similar to the Fig. 3a.

From the analytical solution (2) we can further develop this idea and claim that it is possible to detect the action of microRNA in the "modified" system if one measures the protein synthesis relaxation time: if it significantly increases then microRNA probably acts in the cap-independent manner despite the fact that the steady state rate of the protein synthesis does not change (see the Fig. 3b). *This is a simple consequence of the fact that the relaxation time in a cycle of biochemical reactions is limited by the second slowest reaction *(see [42] or the "Dominant system for a simple irreversible catalytic cycle with limiting step" section in Methods). If the relaxation time is not changed in the presence of microRNA then we can conclude that none of the two alternative mechanisms of microRNA-based translation repression is activated in the system, hence, microRNA action is dependent on the structure of the "wild-type" transcript cap.

The observations from the Fig. 3 are recapitulated in the Table 1. This analysis (of course, over-simplified in many aspects) provides us with an important lesson: observed dynamical features of the translation process with and without presence of microRNA can give clues on the mechanisms of microRNA action and help to distinguish them in a particular experimental situation. Theoretical analysis of the translation dynamics highlights what are the important characteristics of the dynamics which should be measured in order to infer the possible microRNA mechanism.

**Table 1:** Modeling two mechanisms of microRNA action in the simplest linear model

Observable value	Initiation(*k*_1_)	Step after initiation, cap-independent(*k*_2_)	Elongation (*k*_3_)
**Wild-type cap**			
*Steady-state rate*	decreases	decreases	no change
*Relaxation time*	increases slightly	increases slightly	no change
**A-cap**			
*Steady-state rate*	decreases	no change	no change
*Relaxation time*	no change	increases drastically	no change

MicroRNA action effect is described for the protein synthesis steady rate and the relaxation time. It is assumed that the ribosome assembly+elongation step in protein translation, described by the *k*_3 _rate constant, is not rate limiting.

This conclusion suggests the notion of a **kinetic signature of microRNA action mechanism **which we define as *the set of measurable characteristics of the translational machinery dynamics (features of time series for protein, mRNA, ribosomal subunits concentrations) and the predicted tendencies of their changes as a response to microRNA action through a particular biochemical mechanism*.

### The non-linear protein translation model

To explain the effect of microRNA interference with translation initiation factors, a non-linear version of the translation model was proposed [41] which explicitly takes into account recycling of initiation factors (eIF4F) and ribosomal subunits (40S and 60S).

**Figure 4:**
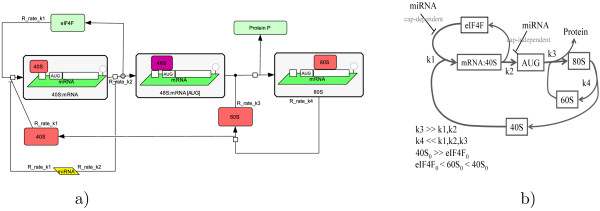
**Non-linear model of microRNA action on the protein translation**. Non-linear model of microRNA action on the protein translation, represented with use of Systems Biology Graphical Notation (a) and schematically with the condition on the constants (b). The difference from the simplest model (Fig. 2) is in the explicit description of initiation factors eIF4F, and ribosomal subunits 40S and 60S recycling.

The model contains the following list of chemical species (see also Fig. 4):

1. 40S, free 40S ribosomal subunit.

2. 60S, free 60S ribosomal subunit.

3. eIF4F, free initiation factor.

4. mRNA:40S, formed initiation complex (containing 40S and the initiation factors), bound to the initiation site of mRNA.

5. AUG, initiation complex bound to the start codon of mRNA.

6. 80S, fully assembled ribosome translating protein.

There are four reactions in the model, all considered to be irreversible:

1. 40S + eIF4F → mRNA:40S, assembly of the initiation complex (rate *k*_1_).

2. mRNA:40S → AUG, some late and cap-independent initiation steps, such as scanning the 5'UTR by for the start codon AUG recognition (rate *k*_2_).

3. AUG → 80S, assembly of ribosomes and protein translation (rate *k*_3_).

4. 80S → 60S+40S, recycling of ribosomal subunits (rate *k*_4_).

The model is described by the following system of equations [41]:(6)

where [40*S*] and [60*S*] are the concentrations of free 40S and 60S ribosomal subunits, [*eIF*4*F*] is a concentration of free translation initiation factors, [*mRNA *: 40*S*] is the concentration of 40S subunit bound to the initiation site of mRNA, [*AUG*] is the concentration of the initiation complex bound to the start codon, [80*S*] is the concentration of ribosomes translating protein, and *Prsynth *is the rate of protein synthesis.

The model (6) contains three independent conservations laws:(7)

The following assumptions on the model parameters were suggested in [41]:(10)

with the following justification: "...The amount 40S ribosomal subunit was set arbitrarily high ... as it is thought to generally not be a limiting factor for translation initiation. In contrast, the level of eIF4F, as the canonical limiting factor, was set significantly lower so translation would be dependent on its concentration as observed experimentally... Finally, the amount of subunit joining factors for the 60S large ribosomal subunit were estimated to be more abundant than eIF4F but still substoichiometric when compared to 40S levels, consistent with in vivo levels... The *k*_4 _rate is relatively slower than the other rates in the model; nevertheless, the simulation's overall protein production was not altered by changes of several orders of magnitude around its value..." [41].

Notice that further in our paper we show that the last statement about the value of *k*_4 _is needed to be made more precise: in the model by Nissan and Parker, *k*_4 _*is a critical parameter *(see "Asymptotology and dynamical limitation theory for biochemical reaction networks" section in Methods). It does not affect the steady state protein synthesis rate only in one of the possible scenarios (inefficient initiation, deficit of the initiation factors).

#### Steady state solution

The final steady state of the system can be calculated from the conservation laws and the balance equations among all the reaction fluxes:(11)

where "s" index stands for the steady state value. Let us designate a fraction of the free [60S] ribosomal subunit in the steady state as . Then we have(12)

and the equation to determine *x*, in which we have neglected the terms of smaller order of magnitude, based on conditions (10):(13)

From the inequalities on the parameters of the model, we have *δ *> 1, *γ *<< 1 and, if *k*_1 _>>*k*_4_/[*eIF*4*F*]_0 _then *α *<<*β*. From these remarks it follows that the constant term *γ*(1 -*β*) of the equation (13) should be much smaller than the other polynomial coefficients, and the equation (13) should have one solution close to zero and two others:(14)

provided that *α *<< |1 - *δ*| or *α *<< |1 - *β|*. In the expression for *x*_1 _we cannot neglect the term proportional to *a*, to avoid zero values in (13).

The solution *x*_2 _is always negative, which means that one can have one positive solution *x*_0 _<< 1 if  and two positive solutions *x*_0 _and *x*_1 _if . However, from (12), (14) and (10) it is easy to check that if *x*_1 _> 0 then *x*_0 _does not correspond to a positive value of [*eIF*4*F*]_*s*_. This means that for a given combination of parameters satisfying (10) we can have only one steady state (either *x*_0 _or *x*_1_).

The two values *x *= *x*_0 _and *x *= *x*_1 _correspond to **two different modes of translation**. When, for example, the amount of the initiation factors [*eIF*4*F*]_0 _is **not enough to provide efficient initiation **(, *x *= *x*_1_) then most of the 40*S *and 60*S *subunits remain in the free form, the initiation factor [*eIF*4*F*] being always the limiting factor. If the **initiation is efficient **enough , then we have *x *= *x*_0 _<< 1 when almost all 60*S *ribosomal subunits are engaged in the protein elongation, and [*eIF*4*F*] being a limiting factor at the early stage, however, is liberated after and ribosomal subunits recycling becomes limiting in the initiation (see the next section for the analysis of the dynamics).

Let us notice that the steady state protein synthesis rate under these assumptions is(15)

This explains the numerical results obtained in [41]: with low concentrations of [*eIF*4*F*]_0 _microRNA action would be efficient only if it affects *k*_2 _or if it competes with *eIF*4*F *for binding to the mRNA cap structure (thus, effectively further reducing the level [*eIF*4*F*]_0_) With higher concentrations of [*eIF*4*F*]_0_, other limiting factors become dominant: [60*S*]_0 _(availability of the heavy ribosomal subunit) and *k*_4 _(speed of ribosomal subunits recycling which is the slowest reaction rate in the system). Interestingly, in any situation the protein translation rate does not depend on the value of *k*_1 _directly (of course, unless it does not become "globally" rate limiting), but only through competing with *eIF*4*F *(which makes the difference with the simplest linear protein translation model).

Equation (15) explains also some experimental results reported in [22]: increasing the concentration of [*eIF*4*F*] translation initiation factor enhances protein synthesis but its effect is abruptly saturated above a certain level.

It would be interesting to make some conclusions on the shift of the polysomal profile from the steady state solutions (14). In this model, the number of ribosomes sitting on mRNA *N*_*polysome *_is defined by , where [*mRNA*] is the concentration of mRNA. However, [*mRNA*] is not an explicit dynamical variable in the model, it is implicitly included in other model constants, such as *k*_1_, together with the effective volume of cytoplasmic space considered in the model. Nevertheless, the model can predict the relative shift of the polysome profile. In the steady state(16)

and *N*_*polysome *_changes in the same way as the protein synthesis steady state value.

#### Analysis of the dynamics

It was proposed to use the following model parameters in [41]: *k*_1 _= *k*_2 _= 2, *k*_3 _= 5, *k*_4 _= 1, [40*S*]_0 _= 100, [60*S*]_0 _= 25, [*eIF*4*F*]_0 _= 6. As we have shown in the previous section, there are two scenarios of translation possible in the Nissan and Parker's model which we called "efficient" and "inefficient" initiation. The choice between these two scenarios is determined by the critical combination of parameters . For the original parameters from [41], *β *= 0.48 < 1 and this corresponds to the simpler one-stage "inefficient" initiation scenario. To illustrate the alternative situation, we changed the value of *k*_4 _parameter, putting it to 0.1, which makes *β *= 4.8 > 1. The latter case corresponds to the "efficient" initiation scenario, the dynamics is more complex and goes in three stages (see below).

**Figure 5:**
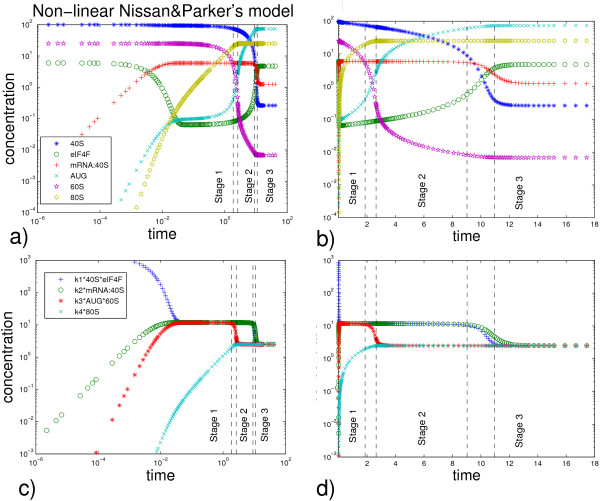
**Simulation of the non-linear protein translation model**. Simulation of the non-linear protein translation model with parameters *k*_1 _= 2, *k*_2 _= 2, *k*_3 _= 5, *k*_4 _= 0.1, [40*S*]_0 _= 100, [60*S*]_0 _= 25, [*eIF*4*F*]_0 _= 6. a) and b) chemical species concentrations at logarithmic and linear scales; c) and d) reaction fluxes at logarithmic and linear scales. By the dashed line several stages are delimited during which the dynamics can be considered as (pseudo-)linear. To determine where ">>" and "<<" conditions are violated, we arbitrarily consider "much bigger" or "much smaller" as difference in one order of magnitude (by factor 10).

Simulations of the protein translation model with these parameters and the initial conditions(17)

are shown on the Fig. 5. The system shows non-trivial relaxation process which takes place in several epochs. Qualitatively we can distinguish the following stages:

1) Stage 1: Relatively fast relaxation with conditions [40*S*] >> [*eIF*4*F*], [60*S*] >> [*AUG*]. During this stage, the two non-linear reactions 40*S *+ *eIF*4*F *→ *mRNA *: 40*S *and *AUG *+ 60*S *→ 80*S *can be considered as pseudo-monomolecular ones: *eIF*4*F *→ *mRNA *: 40*S *and *AUG *→ 80*S *with rate constants dependent on [40*S*] and [60*S*] respectively. This stage is characterized by rapidly establishing quasiequilibrium of three first reactions (R1, R2 and R3 with *k*_1_, *k*_2 _and *k*_3 _constants). Biologically, this stage corresponds to assembling of the translation initiation machinery, scanning for the start codon and assembly of the first full ribosome at the start codon position.

2) Transition between Stage 1 and Stage 2.

3) Stage 2: Relaxation with the conditions [40*S*] >> [*eIF*4*F*], [60*S*] << [*AUG*]. During this stage, the reactions 40*S *+ *eIF*4*F *→ *mRNA *: 40*S *and *AUG *+ 60*S *→ 80*S *can be considered as pseudo-monomolecular *eIF*4*F *→ *mRNA *: 40*S *and 60*S *→ 80*S*. This stage is characterized by two local quasi-steady states established in the two network reaction cycles (formed from R1-R2 and R3-R4 reactions). Biologically, this stage corresponds to the first round of elongation, when first ribosomes moves along the coding region of mRNA. The small ribosomal subunit 40S is still in excess which keeps the initiation stage (reaction R1-R2 fluxes) relatively fast.

4) Transition between Stage 2 and Stage 3.

5) Stage 3: Relaxation with the conditions [40*S*] << [*eIF*4*F*], [60*S*] << [*AUG*]. During this stage, the reactions 40*S *+ *eIF*4*F *→ *mRNA *: 40*S *and *AUG *+ 60*S *→ 80*S *can be considered as pseudo-monomolecular 40*S *→ *mRNA *: 40*S *and 60*S *→ 80*S*. During this stage all reaction fluxes are balanced. Biologically, this stage corresponds to the stable production of the protein with constant recycling of the ribosomal subunits. Most of ribosomal subunits 40S are involved in protein elongation, so the initiation process should wait the end of elongation for that they would be recycled.

**Figure 6:**
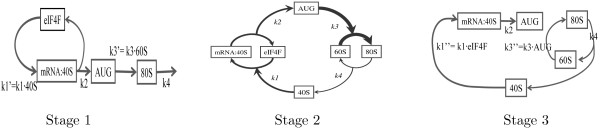
**Dominant systems for three stages of relaxation**. Dominant systems for three stages of relaxation of the model (6). Stage 1) The dominant system is a pseudo-linear network of reactions. Stage 2) The dominant system is a quasi-steady state approximation, where one supposes that the fluxes in two network cycles are balanced. Stage 3) The dominant system is a pseudo-linear network of reactions.

Stages 1-3 can be associated with the corresponding dominant systems [42] which are shown on Fig. 6. Below we give a more detailed analysis of stages 1-3 and transitions between them.

#### Stage 1: translation initiation and assembly of the first ribosome at the start codon

The dominant system of the Stage 1 (Fig. 6a) can be modeled as a linear system of equations (notice that it is not equivalent to the system of equations that would correspond to fully monomolecular reaction network because the reaction R2 is still bimolecular despite the fact that the products of this reaction do not interact, which leads to the linear description):(18)

where  = *k*_1 _· [40*S*],  = *k*_3 _· [60*S*] and we consider that at this stage the changes of 40S and 60S are relatively slow. This system has simple approximate solution, taking into account constraints on the parameters *k*_2 _<<, ; *k*_4 _<<, , *k*_2_, also assuming *k*_2 _<< | - |, and for the initial condition(19)

we have(20)

From this solution, one can conclude that the relaxation of this model goes at several time scales (very rapid *~ min*(1/, 1/) and slow ~1/*k*_4_) and that when eIF4F, mRNA:40S and AUG already reached their quasiequilibrium values, [80S] continues to grow. This corresponds to the quasiequilibrium approximation asymptotics (see the "Quasi steady-state and quasiequilibrium asymptotics" section of the Methods). At some point 80S will reach such a value that it would be not possible to consider 60S constant: otherwise the conservation law (9) will be violated. This will happen when [80*S*] << [60*S*] condition is not satisfied anymore, i.e., following our convention to consider "much smaller" as difference in one order of magnitude, at . The same consideration is applicable for another conservation law (7) in which [80*S*] is included, but from the time point . From [40*S*]_0 _>> [*eIF*4*F*]_0 _and [40*S*]_0 _> [60*S*]_0 _we have *min*(*t', t"*) = *t'*. This means that the parameters [40*S*], [60*S*] of the (local) steady states for [*eIF*4*F*] and [*AUG*] should slowly (at the same rate as [80*S*]) change from the time point *t' *(variable [*mRNA: *40*S*] does not change because its local steady state does not depend on [40*S*], [60*S*]). In other words, after *t *= *t' *the Stage 1 solution (20) should be prolonged as(21)

From these equations, one can determine the effective duration of the Stage 1: by definition, it will be finished when one of the two conditions ([40*S*] >> [*eIF*4*F*], [60*S*] >> [*AUG*]) will be violated, which happens at times ~  and ~  correspondingly, hence, the second condition will be violated first (from [60*S*]_0 _< [40*S*]_0_).

#### Stage 2: first stage of protein elongation, initiation is still rapid

The Stage 2 is characterized by conditions [*eIF*4*F*] << [40*S*], [60*S*] << [*AUG*]. This fact can be used for deriving the quasi-steady state approximation: we assume that the reaction fluxes in two network cycles (R1-R2 and R3-R4) are independently balanced:(22)

Then (6) is simplified and, using the conservation laws, we have a single equation on [40*S*]:(23)

where *A *= [40*S*](*t*) + [*AUG*](*t*) is a constant quantity conserved accordingly to the quasi-steady state approximation (see "Quasi steady-state and quasiequilibrium asymptotics" section of Methods). Equation (23) can be already integrated but let us further simplify it for our analysis. Having in mind *k*_4 _<<*k*_3 _and assuming that at the beginning of the Stage 2 [*AUG*] >>, we can simplify (23) to(24)

and, further, assuming that at the beginning of the Stage 2 we have [40*S*] >> [*AUG*] let us approximate the right-hand side of the equation by a piecewise-linear function(25)

where [40*S*]|_*t*=*t" *_is the amount of 40*S *at the beginning of the Stage 2. Then the descent of [40*S*](*t*) can be separated into linear and exponential phases:(26)

where *K*_1_, *K*_2 _are linear and exponential slopes and [40*S*]_*s*2 _is the quasi-steady state value of [40*S*] at the end of the Stage 2:(27)

Other dynamic variables are expressed through [40*S*](*t*) as(28)

At some point, the amount of free small ribosomal subunit 40S, which is abundant at the beginning of the Stage 2, will not be sufficient to support rapid translation initiation. Then the initiation factor *eIF*4*F *will not be the limiting factor in the initiation and the condition [40*S*] >> [*eIF*4*F*] will be violated. We can estimate this time as .

#### Stage 3: steady protein elongation, speed of initiation equals to speed of elongation

During the Stage 3 all fluxes in the network become balanced and the translation arrives at the steady state. From Fig. 6 it is clear that the relaxation goes independently in the cycle *R*3 - *R*4, where the relaxation equations are simply(29)

where *t"' *is the time when the Stage 3 of the relaxation starts. This relaxation goes relatively fast, since *k*_3_[*AUG*]|_*t*=*t''' *_is relatively big. So, during the Stage 3, one can consider the cycle *R*1 - *R*4 equilibrated, with [80*S*] = [80]_*s*_, [60*S*] = [60]_*s *_values.

Hence, the relaxation during the Stage 3 consists in redistributing concentrations of 40S and mRNA:40S to their steady states in a linear chain of reactions *R*1 - *R*2 (the value of [*AUG*] is relatively big and can be adjusted from the conservation law (7)). Using the pseudo-linear approximation of this stage (see Fig. 6), we can easily write down the corresponding approximate relaxation equations:(30)

where *B *= (1 - ) ([40]_*s*_- [40*S*]|_*t *= *t'''*_). [40]_*s *_and [*mRNA *: 40*S*]_*s *_are the steady-state values of the corresponding variables, see (12). The values [60*S*]|_*t *= *t'''*, _[*eIF*4*F*]|_*t *= *t'''*, _[*AUG*]|_*t *= *t''' *_and [*mRNA *: 40*S*]|_*t *= *t*″' _can be estimated from (28), using the [40*S*]|_*t *= *t''' *_value. The relaxation time at this stage equals

The solution for the Stage 3 can be further simplified if *k*_2 _<<*k*_1_[*eIF*4*F*]|_*t *= *t''' *_or *k*_2 _>>*k*_1 _[*eIF*4*F*]|_*t *= *t*'''_.

#### Transitions between stages

Along the trajectory of the dynamical system (6) there are three dominant system each one transforming into another. At the transition between stages, two neighbor dominant systems are united and then split. Theoretically, there might be situations when the system can stay in these transition zones for long periods of time, even infinitely. However, in the model (6) this is not the case: the trajectory rapidly passes through the transition stages and jumps into the next dominant system approximation.

Three dominant approximations can be glued, using the concentration values at the times of the switching of dominant approximation as initial values for the next stage. Note that the Stage 2 has essentially one degree of freedom since it can be approximated by a single equation (23). Hence, one should only know one initial value [40*S*]|_*t *= *t'' *_to glue the Stages 1 and 2. The same is applied to the gluing of Stages 2 and 3, since in the end of Stage 2 all variable values are determined by the value of [40*S*]|_*t *= *t'''*_.

#### Case of always limiting initiation

As it follows from our analysis, the most critical parameter of the non-linear protein translation model is the ratio . Above we have considered the case *β *> 1 which is characterized by a switch of the limiting factor in the initiation (from *eIF*4*F *at the Stages 1 and 2 to 40*S *at the Stage 3).

**Figure 7:**
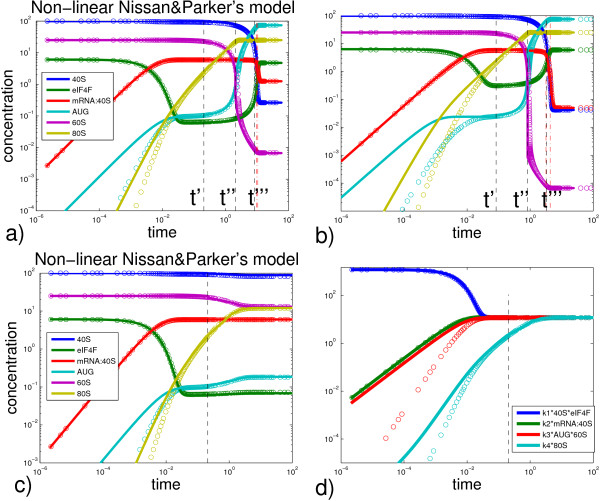
**Comparison of the numerical and approximate analytical solutions of the non-linear protein translation model**. Examples of the exact numerical (circles) and approximate analytical (solid lines) solutions of the non-linear protein translation model. a) For the set of parameters *k*_1 _= 2, *k*_2 _= 2, *k*_3 _= 5, *k*_4 _= 0.1; b) For parameters *k*_1 _= 1, *k*_2 _= 5, *k*_3 _= 50, *k*_4 _= 0.01; c) For the set of parameters from [41], *k*_1 _= 2, *k*_2 _= 2, *k*_3 _= 5, *k*_4 _= 1; d) Reaction fluxes for the set of parameters c). Dashed black vertical lines denote evaluated transition points between the dynamics stages. Dashed red vertical points denote the time points where [40*S*](*t*) = 10·[*eIF*4*F*](*t*) and [40*S*](*t*) = [*eIF*4*F*](*t*)/10 respectively.

In the case *β *< 1 the dynamics becomes simpler and consists of one single stage: relaxation accordingly to (20) and further with correction (21) with the relaxation time ~  (the quasiequilibrium approximation corresponding to the Stage 1 works well for the whole translation process). The reason for this is that if the initiation is not efficient then the system is never in the situation of the Stage 2 conditions when the cycle R1-R2 is balanced with much bigger flux than the cycle R3-R4. This approximation is the more exact the smaller *β *value, however, the value of *β *should not be necessary very small. For example, for the default parameter values of the model *β *= 0.48, and it well reproduces the dynamics (see Fig. 7c-d). From numerical experiments one can see that even for *β *= 0.95 the dynamics is qualitatively well reproduced. To model the A-cap structure effect with very weak capacity for initiation (assembly of the initiation factors and 40S subunit), we should also consider the case(31)

for which the solution derived above is not directly applicable. However, the analytical calculations in this case can be performed in the same fashion as above. The detailed derivation of the solution is given in Additional file 1. The effect of putting *k*_1 _very small on the steady state protein synthesis and the relaxation time is shown on Fig. 7.

In a similar way all possible solutions of the equations (6) with very strong inhibitory effect of microRNA on a particular translation step can be derived. These solutions will describe the situation when the effect of microRNA is so strong that it changes the dominant system (limiting place of the network) by violating the initial constraints (10) on the parameters (for example, by making *k*_3 _smaller than other *k*_*i*_s). Such possibility exists, however, it can require too strong (non-physiological) effect of microRNA-dependent translation inhibition.

#### Effect of microRNA on the translation dynamics

Our analysis of the non-linear Nissan and Parker's model showed that the protein translation machinery can function in two qualitatively different modes, determined by the ratio . We call these two modes "efficient initiation" (*β *> 1) and "inefficient initiation" (*β *< 1) scenarios. Very roughly, this ratio determines the balance between the overall speeds of initiation and elongation processes. In the case of "efficient initiation" the rate of protein synthesis is limited by the speed of recycling of the ribosomal components (60S). In the case of "inefficient initiation" the rate of protein synthesis is limited by the speed of recycling of the initiation factors (eIF4F). Switching between two modes of translation can be achieved by changing the availability of the corresponding molecules ([60*S*]_0 _or [*eIF*4*F*]_0_) or by changing the critical kinetic parameters (*k*_2 _or *k*_4_). For example, changing *k*_4 _from 1 (Fig. 7c) to 0.1 (Fig. 7a), performs such a switch for the original parameter values from [41].

As a result of the dynamical analysis, we can assemble an approximate solution of the non-linear system under assumptions (10) about the parameters. An example of the approximate solution is given on Fig. 7. The advantage of such a semi-analytical solution is that one can predict the effect of changing the system parameters. For example, on Fig. 7b the solution is compared to an exact numerical one, where the parameters have been changed but still obey the initial constraints (10).

One of the obvious predictions is that the dynamics of the system is not sensitive to variations of *k*_3_, so if microRNA acts on the translation stage controlled by *k*_3 _then no microRNA effect could be observed looking at the system dynamics (being the fastest one, *k*_3 _is not a critical parameter in any scenario).

**Figure 8:**
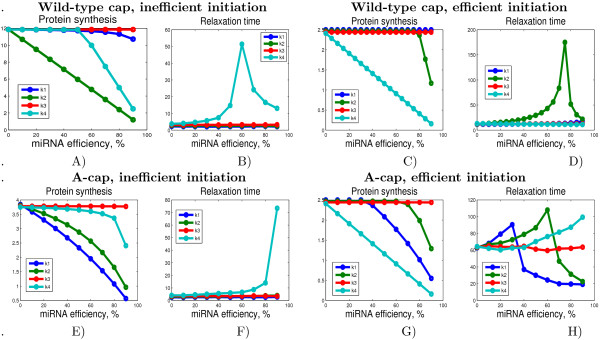
**Effect of mimicking different mechanisms of miRNA action on translation**. Effect of decreasing some model parameters mimicking different mechanisms of miRNA action on translation. Relaxation time here is defined as the latest time at which any chemical species in the model differs from its final steady state by 10% A) and B) correspond to the scenario with "inefficient" initiation, with use of the model parameters proposed in [41] (*k*_1 _= *k*_2 _= 2, *k*_3 _= 5, *k*_4 _= 1, [*eIF*4*F*]_0 _= 6, [60*S*]_0 _= 25, [40*S*]_0 _= 100), which gives *β *= 0.48 < 1. C) and D) correspond to the scenario with "efficient" initiation, with parameters (*k*_1 _= 2, *k*_2 _= 3, *k*_3 _= 50, *k*_4 _= 0.1, [*eIF*4*F*]_0 _= 6, [60*S*]_0 _= 25, [40*S*]_0 _= 100), which gives *β *= 7.2 > 1. The absciss value indicates the degree of inhibition (decreasing) of a parameter. E-H) same as A-D) but for a modified cap structure, modeled by reduced *k*_1 _parameter: *k*_1 _= 0.01 for these curves, the other parameters are the same as on A-D) correspondingly.

If microRNA acts on the translation stage controlled by *k*_4 _(for example, by ribosome stalling mechanism) then we should consider two cases of efficient (*β *> 1) and inefficient (*β *< 1) initiation. In the first case the steady state protein synthesis rate is controlled by *k*_4 _(as the slowest, limiting step) and any effect on *k*_4 _would lead to the proportional change in the steady state of protein production. By contrast, in the case of inefficient initiation, the steady state protein synthesis is not affected by *k*_4_. Instead, the relaxation time is affected, being ~ . However, diminishing *k*_4 _increases the *β *parameter, hence, this changes "inefficient initiation" scenario for the opposite, hence, making *k*_4 _critical for the steady state protein synthesis anyway when *k*_4 _becomes smaller than . For example, for the default parameters of the model, decreasing *k*_4 _value firstly leads to no change in the steady state rate of protein synthesis, whereas the relaxation time increases and, secondly, after the threshold value  starts to affect the steady state protein synthesis rate directly (see Fig. 8A, B). This is in contradiction to the message from [41] that the change in *k*_4 _by several orders of magnitude does not change the steady state rate of protein synthesis.

Analogously, decreasing the value of *k*_2 _can convert the "efficient" initiation scenario into the opposite after the threshold value . We can recapitulate the effect of decreasing *k*_2 _in the following way. 1) in the case of "efficient" initiation *k*_2 _does not affect the steady state protein synthesis rate up to the threshold value after which it affects it in a proportional way. The relaxation time drastically increases, because decreasing *k*_2 _leads to elongation of all dynamical stages duration (for example, we have estimated the time of the end of the dynamical Stage 2 as ). However, after the threshold value the relaxation time decreases together with *k*_2_, quickly dropping to its unperturbed value (see Fig. 8C-D). 2) in the case of "inefficient" initiation the steady state protein synthesis rate depends proportionally on the value of *k*_2 _(15), while the relaxation time is not affected (see Fig. 8A-B).

MicroRNA action on *k*_1 _directly does not produce any strong effect neither on the relaxation time nor on the steady state protein synthesis rate. This is why in the original work [41] cap-dependent mechanism of microRNA action was taken into account through effective change of the [*eIF*4*F*]_0 _value (total concentration of the translation initiation factors), which is a critical parameter of the model (see 15).

The effect of microRNA on various mechanism and in various experimental settings (excess or deficit of eIF4F, normal cap or A-cap) is recapitulated in Table 2. The conclusion that can be made from this table is that all four mechanisms show clearly different patterns of behavior in various experimental settings. From the simulations one can make a conclusion that it is still not possible to distinguish between the situation when microRNA does not have any effect on protein translation and the situation when it acts on the step which is neither rate limiting nor "second rate limiting" in any experimental setting (*k*_3 _in our case). Nevertheless, if any change in the steady-state protein synthesis or the relaxation time is observed, theoretically, it will be possible to specify the mechanism responsible for it.

**Table 2:** Modeling of four mechanisms of microRNA action in the non-linear protein translation model

Observable value	Initiation(*k*_1_)	Step after initiation(*k*_2_)	Ribosome assembly (*k*_3_)	Elongation (*k*_4_)
**Wild-type cap, inefficient initiation**				
*Steady-state rate*	slightly decreases	decreases	no change	decreases after threshold
*Relaxation time*	no change	no change	no change	goes up and down
**Wild-type cap, efficient initiation**				
*Steady-state rate*	no change	slightly decreases after strong inhibition	no change	decreases
*Relaxation time*	no change	goes up and down	no change	no change
**A-cap, inefficient initiation**				
*Steady-state rate*	decreases	decreases	no change	slightly decreases after strong inhibition
*Relaxation time*	no change	no change	no change	goes up and down
**A-cap, efficient initiation**				
*Steady-state rate*	decreases after threshold	slightly decreases after strong inhibition	no change	decreases
*Relaxation time*	goes up and down	goes up and down	no change	increases

MicroRNA action effect is described for the protein synthesis steady rate and the relaxation time (see also Fig. 8). "Efficient initiation" and "inefficient initiation" correspond to two qualitatively different solution types of Nissan and Parker's model (see the beginning of "Effect of microRNA on the translation dynamics" section). The effect of A-cap structure is modeled by making the cap-dependent initiation step very slow (by making the *k*_1 _parameter very small). It is assumed that the ribosome assembly step in protein translation, described by the *k*_3 _rate constant, is not rate limiting.

### Available experimental data and possible experimental validation

It is important to underline that the Nissan and Parker's models analyzed in this paper are qualitative descriptions of the protein translation machinery. The parameter values used represent rough order-of-magnitude estimations or real kinetic rates. Moreover, these values should be considered as relative and unitless since they do not match any experimental time scale (see below). Nevertheless, such qualitative description already allows to make predictions on the relative changes of the steady states and relaxation times (see the Table 2), and in principle these predictions can be verified experimentally. Let us imagine an experiment in which it would be possible to verify such predictions. In this experiment, two time series should be compared: 1) one measured in a system in which microRNA acts on a normal "wild-type" protein translation machinery and 2) another system almost fully identical to the first one but in which one of the translation stages is modified (made slow and rate-limiting, or, opposite, very rapid). There are multiple possibilities to modify the rate of this or that translation stage. The initiation can be affected by changing the concentration of the initiation factors such as eIF4F as in [22]. The scanning stage can be affected by introducing various signals in the 5'UTR sequence of mRNA such as in-frame AUG codons (see, for example, [43]). In principle, the elongation stage can be modified by introducing slow synonymous codons in the coding sequence (there even exist mathematical models of their effect [33,35,37] that can be used for the optimal experiment design). The stage of elongation termination can be influenced by varying the concentration of the corresponding release factors (ETF1 or ETF2), at least *in vitro*. The two time series measured after activation or introduction of microRNA should be characterized for the relative changes of steady state values and relaxation times of protein and mRNA concentrations, and, if possible, the number of ribosomes in the polysome. Also, ideally, it is desired to construct several experimental systems in which the amount of inhibition by microRNA can be gradually changed (for example, by changing the number of the corresponding seed sequences in the 3'UTR region).

To the best of our knowledge, there is no such a dataset published until so far, even partially. In several recent papers, one can find published time series of protein and mRNA concentrations or their relative changes measured after introducing microRNA. For example, the deadenylation time course is shown in [25]: translation decreases after 20 min and stops at 30 min, deadenylation begin at 30 min, goes around 1 h. In [44], the authors study the kinetics of degradation of mRNA. After adding microRNA to the system, the amount and the length of the targeted mRNA starts to decrease at around 3-5 hours, and decreases by 90% at 8 hours. In [45], the authors study the global change of protein after transfection of a microRNA. They described a small change at the mRNA level at 8 h after miRNA transfection, and the considerable decrease appeared only after 32 hours while the protein concentration change was apparent at the time-course between 8 hours and 32 hours. In the *in vitro *system used in [22], at 15 min after incubation with microRNA there was already a 25% decrease of translation, indicating that the translational inhibition can be a relatively rapid mechanism.

These data on protein translation kinetics show that the relaxation time range could vary from several minutes to several hours and even tens of hours depending on the critical step affected, on various mRNA properties and on the whole biological system taken for the experiment (for example, the presence or absence of different effectors influencing different steps of the translation process). These data should be taken into account when constructing more realistic and quantitative models of microRNA action on protein translation.

## Discussion

The role of microRNA in gene expression regulation is discovered and confirmed since ten years, however, there is still a lot of controversial results regarding the role of concrete mechanisms of microRNA-mediated protein synthesis repression. Some authors argue that it is possible that the different modes of microRNA action reflect different interpretations and experimental approaches, but the possibility that microRNAs do indeed silence gene expression via multiple mechanisms also exists. Finally, microRNAs might silence gene expression by a common and unique mechanism; and the multiple modes of action represent secondary effects of this primary event [1-3].

The main reason for accepting a possible experimental bias could be the studies in vitro, where conditions are strongly different from situation in vivo. Indeed, inside the cell, mRNAs (microRNA targets) exist as ribonucleoprotein particles or mRNPs, and second, all proteins normally associated with mRNAs transcribed in vivo are absent or at least much different from that bound to the same mRNA in an in vitro system or following the microRNAs transfection into cultured cells. The fact that RNA-binding proteins strongly influence the final outcome of microRNA regulation is proved now by several studies [19,46,47]. The mathematical results provided in this paper suggests a complementary view on the co-existence of multiple microRNA-mediated mechanisms of translation repression. Mathematical modeling suggests to us to ask a question: *if multiple mechanisms act simultaneously, would all of them equally contribute to the final observable repression of protein synthesis or its dynamics? *The dynamical limitation theory gives an answer: *the effect of microRNA action will be observable and measurable in two cases: 1) if it affects the dominant system of the protein translationary machinery, or 2) if the effect of microRNA action is so strong that it changes the limiting place (the dominant system)*.

In a limited sense, this means, in particular, that the protein synthesis steady rate is determined by the limiting step in the translation process and any effect of microRNA will be measurable only if it affects the limiting step in translation, as it was demonstrated in [41]. Due to the variety of external conditions, cellular contexts and experimental systems the limiting step in principle can be any in the sequence of events in protein translation, hence, this or that microRNA mechanism can become dominant in a concrete environment. However, when put on the language of equations, the previous statement already becomes non-trivial in the case of non-linear dynamical models of translation (and even linear reaction networks with non-trivial network structure). Our analysis demonstrates that the limiting step in translation can change with time, depends on the initial conditions and is not represented by a single reaction rate constant but rather by some combination of several model parameters. Methodology of dynamical limitation theory that we had developed [42,48], allows to deal with these situations on a solid theoretical ground.

Furthermore, in the dynamical limitation theory, we generalize the notion of the limiting step to the notion of dominant system, and this gives us a possibility to consider not only the steady state rate but also some dynamical features of the system under study. One of the simplest measurable dynamical feature is the *protein synthesis relaxation time*, i.e. the time needed for protein synthesis to achieve its steady state rate. The general idea of "relaxation spectrometry" goes back to the works of Manfred Eigen, a Nobel laureate [49] and is still underestimated in systems biology. Calculation of the relaxation time (or times) requires careful analysis of time scales in the dynamical system, which is greatly facilitated by the recipes proposed in [42,50]. As we have demonstrated in our semi-analytical solutions, measuring the steady state rate and relaxation time at the same time allows to detect which step is possibly affected by the action of microRNA (resulting in effective slowing down of this step). To our knowledge, this idea was never considered before in the studies of microRNA-dependent expression regulation. The Table 2 recapitulates predictions allowing to discriminate a particular mechanism of microRNA action.

## Conclusions

The analysis of the transient dynamics gives enough information to verify or reject a hypothesis about a particular molecular mechanism of microRNA action on protein translation. For multiscale systems only that action of microRNA is distinguishable which affects the parameters of dominant system (critical parameters), or changes the dominant system itself. Dominant systems generalize and further develop the old and very popular idea of limiting step. Algorithms for identifying dominant systems in multiscale kinetic models are straightforward but not trivial and depend only on the ordering of the model parameters but not on their concrete values. Asymptotic approach to kinetic models of biological networks suggests new directions of thinking on a biological problem, making the mathematical model a useful tool accompanying biological reasoning and allowing to put in order diverse experimental observations.

However, to convert the methodological ideas presented in this paper into a working tool for experimental identification of the mechanisms of microRNA-dependent protein translation inhibition, requires special efforts. Firstly, we need to construct a model which would include all known mechanisms of microRNA action. Secondly, realistic estimations on the parameter value intervals should be made. Thirdly, careful analysis of qualitatively different system behaviors should be performed and associated with the molecular mechanisms. Fourthly, a critical analysis of available quantitative information existing in the literature should be made. Lastly, the experimental protocols (sketched in the previous section) for measuring dynamical features such as the relaxation time should be developed. All these efforts makes a subject of a separate study which is an ongoing work.

## Methods

### Asymptotology and dynamical limitation theory for biochemical reaction networks

Most of mathematical models that really work are simplifications of the basic theoretical models and use in the backgrounds an assumption that some terms are big, and some other terms are small enough to neglect or almost neglect them. The closer consideration shows that such a simple separation on "small" and "big" terms should be used with precautions, and special culture was developed. The name "asymptotology" for this direction of science was proposed by [51] defined as "the art of handling applied mathematical systems in limiting cases".

In chemical kinetics three fundamental ideas were developed for model simplification: *quasiequilibrium asymptotic *(QE), *quasi steady-state asymptotic *(QSS) and the idea of *limiting step*.

In the IUPAC Compendium of Chemical Terminology (2007) one can find **a definition of limiting step **[52]: "A rate-controlling (rate-determining or rate-limiting) step in a reaction occurring by a composite reaction sequence is an elementary reaction the rate constant for which exerts a strong effect -stronger than that of any other rate constant - on the overall rate."

Usually when people are talking about limiting step they expect significantly more: there exists a rate constant which exerts such a strong effect on the overall rate that the effect of all other rate constants together is significantly smaller. For the IUPAC Compendium definition a rate-controlling step always exists, because among the control functions generically exists the biggest one. On the contrary, for the notion of limiting step that is used in practice, there exists a difference between systems with limiting step and systems without limiting step.

During XX century, the concept of the limiting step was revised several times. First simple idea of a "narrow place" (the least conductive step) could be applied without adaptation only to a simple cycle or a chain of irreversible steps that are of the first order (see Chap. 16 of the book [53] or [54] or the section "Dominant system for a simple irreversible catalytic cycle with limiting step" of this paper). When researchers try to apply this idea in more general situations they meet various difficulties.

Recently, we proposed a new theory of dynamic and static limitation in multiscale reaction networks [42,48]. This approach allows to find the simplest network which can substitute a multiscale reaction network such that the dynamics of the complex network can be approximated by the simpler one. Following the asymptotology terminology [55], we call this simple network the **dominant system **(DS). In the simplest cases, the dominant system is a subsystem of the original model. However, in the general case, it also includes new reactions with kinetic rates expressed through the parameters of the original model, and rates of some reactions are renormalized: hence, *in general, the dominant system is not a subsystem of the original model*.

The dominant systems can be used for direct computation of steady states and relaxation dynamics, especially when kinetic information is incomplete, for design of experiments and mining of experimental data, and could serve as a robust first approximation in perturbation theory or for preconditioning.

*Dominant systems serve as correct generalization of the limiting step notion in the case of complex multiscale networks*. They can be used to answer an important question: given a network model, which are its critical parameters? Many of the parameters of the initial model are no longer present in the dominant system: these parameters are **non-critical**. Parameters of dominant systems (**critical parameters**) indicate putative targets to change the behavior of the large network.

Most of reaction networks are nonlinear, it is nevertheless useful to have an efficient algorithm for solving linear problems. First, nonlinear systems often include linear subsystems, containing reactions that are (pseudo)monomolecular with respect to species internal to the subsystem (at most one internal species is reactant and at most one is product). Second, for binary reactions *A *+ *B *→ ..., if concentrations of species *A *and *B *(*c*_*A*_, *c*_*B*_) are well separated, say *c*_*A *_>>*c*_*B *_then we can consider this reaction as *B *→ ... with rate constant proportional to *c*_*A *_which is practically constant, because its relative changes are small in comparison to relative changes of *c*_*B*_. We can assume that this condition is satisfied for all but a small fraction of genuinely non-linear reactions (the set of non-linear reactions changes in time but remains small). Under such an assumption, non-linear behavior can be approximated as a sequence of such systems, followed one each other in a sequence of "phase transitions". In these transitions, the order relation between some of species concentrations changes. Some applications of this approach to systems biology are presented by [50]. The idea of controllable linearization "by excess" of some reagents is in the background of the efficient experimental technique of Temporal Analysis of Products (TAP), which allows to decipher detailed mechanisms of catalytic reactions [56].

Below we give some details on the approaches used in this paper to analyze the models by Nissan and Parker [41].

### Notations

To define a chemical reaction network, we have to introduce:

• a list of components (species);

• a list of elementary reactions;

• a kinetic law of elementary reactions.

The list of components is just a list of symbols (labels) *A*_1_,...*A*_*n*_. Each elementary reaction is represented by its *stoichiometric equation*(32)

where *s *enumerates the elementary reactions, and the non-negative integers *α*_*si*_, *β*_*si *_are the *stoichiometric coefficients*. A stoichiometric vector *γ*_*s *_with coordinates. *γ*_*si *_= *β*_*si*_- *α*_*si *_is associated with each elementary reaction.

A non-negative real *extensive *variable *N*_*i *_≥ 0, amount of *A*_*i*_, is associated with each component *A*_*i*_. It measures "the number of particles of that species" (in particles, or in moles). The concentration of *A*_*i *_is an *intensive *variable: *c*_*i *_= *N*_*i*_/*V*, where *V *is volume. In this paper we consider the volume (of cytoplasm) to be constant. Then the kinetic equations have the following form(33)

where *T *is the temperature, *w*_*s *_is the rate of the reaction *s*, *v *is the vector of external fluxes normalized to unite volume. It may be useful to represent external fluxes as elementary reactions by introduction of new component Ø together with income and outgoing reactions Ø → *A*_*i *_and *A*_*i *_→ Ø.

The most popular *kinetic law *of elementary reactions is the *mass action law *for perfect systems:(34)

where *k*_*s *_is a "kinetic constant" of the reaction *s*.

### Quasi steady-state and quasiequilibrium asymptotics

*Quasiequilibrium approximation uses the assumption that a group of reactions is much faster than other *and goes fast to its equilibrium. We use below superscripts '^f^' and '^s^' to distinguish fast and slow reactions. A small parameter appears in the following form(35)

To separate variables, we have to study the spaces of linear conservation law of the initial system (35) and of the fast subsystem

If they coincide, then the fast subsystem just dominates, and there is no fast-slow separation for variables (all variables are either fast or constant). But if there exist additional linearly independent linear conservation laws for the fast system, then let us introduce new variables: linear functions *b*^1^(*c*),...*b*^*n*^(*c*), where *b*^1^(*c*),...*b*^*m*^(*c*) is the basis of the linear conservation laws for the initial system, and *b*^1^(*c*),...*b*^*m*+*l*^(*c*) is the basis of the linear conservation laws for the fast subsystem. Then *b*^*m*+*l*+1^(*c*),...*b*^*n*^(*c*) are fast variables, *b*^*m*+1^(*c*),...*b*^*m*+*l*^(*c*) are slow variables, and *b*^1^(*c*),...*b*^*m*^(*c*) are constant. The *quasiequilibrium manifold *is given by the equations  and for small *ε *it serves as an approximation to a slow manifold.

The **quasi steady-state **(or pseudo steady state) assumption was invented in chemistry for description of systems with radicals or catalysts. In the most usual version the species are split in two groups with concentration vectors *c*^s ^("slow" or basic components) and *c*^f ^("fast intermediates"). For catalytic reactions there is additional balance for *c*^f^, amount of catalyst, usually it is just a sum *The amount of the fast intermediates is assumed much smaller than the amount of the basic components*, but the reaction rates are of the same order, or even the same (both intermediates and slow components participate in the same reactions). This is the source of a small parameter in the system. Let us scale the concentrations *c*^f ^and *c*^s ^to the compatible amounts. After that, the fast and slow time appear and we could write *c*^s ^= *W*^s^(*c*^s^, *c*^f^), , where *ε *is small parameter, and functions *W*^s^, *W*^f ^are bounded and have bounded derivatives (are "of the same order"). We can apply the standard singular perturbation techniques. If dynamics of fast components under given values of slow concentrations is stable, then the slow attractive manifold exists, and its zero approximation is given by the system of equations *W*^f^(*c*^s^, *c*^f^) = 0.

The QE approximation is also extremely popular and useful. It has simpler dynamical properties (respects thermodynamics, for example, and gives no critical effects in fast subsystems of closed systems).

Nevertheless, neither radicals in combustion, nor intermediates in catalytic kinetics are, in general, close to quasiequilibrium. They are just present in much smaller amount, and when this amount grows, then the QSS approximation fails.

The simplest demonstration of these two approximation gives the simple reaction: *S *+ *E *↔ *SE *→ *P *+ *E *with reaction rate constants  and *k*_2_. The only possible quasiequilibrium appears when the first equilibrium is fast:  = *κ*^±^/*ε*. The corresponding slow variable is *C*^*s *^= *c*_*S *_+ *c*_*SE*_, *b*_*E *_= *c*_*E *_+ *c*_*SE *_= *const*.

For the QE manifold we get a quadratic equation . This equation gives the explicit dependence *C*_*SE*_(*C*^*s*^), and the slow equation reads *Ċ*^*s *^= -*k*_2_*c*_*SE*_(*C*_*s*_), *C*^*s *^+ *c*_*P *_= *b*_*S *_= *const*.

For the QSS approximation of this reaction kinetics, under assumption *b*_*E*_<<*b*_*S*_, we have fast intermediates *E *and *SE*. For the QSS manifold there is a linear equation , which gives us the explicit expression for *c*_*SE*_(*c*_*S*_):  (the standard Michaelis-Menten formula). The slow kinetics reads . The difference between the QSS and the QE in this example is obvious.

The terminology is not rigorous, and often QSS is used for all singular perturbed systems, and QE is applied only for the thermodynamic exclusion of fast variables by the maximum entropy (or minimum of free energy, or extremum of another relevant thermodynamic function) principle (MaxEnt). This terminological convention may be convenient. Nevertheless, without any relation to terminology, the difference between these two types of introduction of a small parameter is huge. There exists plenty of generalizations of these approaches, which aim to construct a slow and (almost) invariant manifold, and to approximate fast motion as well. The following references can give a first impression about these methods: Method of Invariant Manifolds (MIM) ([57,58], Method of Invariant Grids (MIG), a discrete analogue of invariant manifolds ([59]), Computational Singular Perturbations (CSP) ([60-62]) Intrinsic Low-Dimensional Manifolds (ILDM) by [63], developed further in series of works by [64]), methods based on the Lyapunov auxiliary theorem ([65]).

### Multiscale monomolecular reaction networks

*A monomolecular reaction is such that it has at most one reactant and at most one product*. Let us consider a general network of monomolecular reactions. This network is represented as a directed graph (digraph) [66]: vertices correspond to components *A*_*i*_, edges correspond to reactions *A*_*i *_→ *A*_*j *_with kinetic constants *k*_*ji *_> 0. For each vertex, *A*_*i*_, a positive real variable *c*_*i *_(concentration) is defined.

"Pseudo-species" (labeled Ø) can be defined to collect all degraded products, and degradation reactions can be written as *A*_*i *_→ Ø with constants *k*_0*i*_. Production reactions can be represented as Ø → *A*_*i *_with rates *k*_*i*0_. The kinetic equation for the system is(36)

or in vector form: ċ = *K*_0 _+ *Kc*. Solution of this system can be reduced to a linear algebra problem: let us find all left (*l*^*i*^) and right (*r*^*i*^) eigenvectors of *K*, i.e.:(37)

with the normalization (*l*^*i*^, *r*^*i*^) = *δ*_*ij*_, where *δ*_*ij *_is Kronecker's delta. Then the solution of (36) is(38)

where *c*^*s *^is the steady state of the system (36), i.e. when all  = 0, and *c*(0) is the initial condition.

If all reaction constants *k*_*ij *_would be known with precision then the eigenvalues and the eigenvectors of the kinetic matrix can be easily calculated by standard numerical techniques. Furthermore, Singular Value Decomposition (SVD) can be used for model reduction. But in systems biology models often one has only approximate or relative values of the constants (information on which constant is bigger or smaller than another one). Let us consider the simplest case: when all kinetic constants are very different (separated), i.e. for any two different pairs of indices *I *= (*i, j*), *J *= (*i', j'*) we have either *k*_*I *_>>*k*_*J *_or *k*_*J *_<<*k*_*I*_. In this case we say that the system is **hierarchical ***with timescales *(inverses of constants *k*_*ij*_, *j *≠ 0) *totally separated*.

Linear network with totally separated constants can be represented as a digraph and a set of orders (integer numbers) associated to each arc (reaction). The lower the order, the more rapid is the reaction. It happens that in this case the special structure of the matrix *K *(originated from a reaction graph) allows us to exploit the strong relation between the dynamics (36) and the topological properties of the digraph. In this case, possible values of *l*_*i *_k are 0, 1 and the possible values of *r*_*i *_are -1, 0, 1 with high precision. In previous works, we provided an algorithm for finding non-zero components of *l*_*i*_, *r*_*i*_, based on the network topology and the constants ordering, which gives a good approximation to the problem solution [42,48,50].

### Dominant system for a simple irreversible catalytic cycle with limiting step

A linear chain of reactions, *A*_1 _→ *A*_2 _→ ...*A*_*n*_, with reaction rate constants *k*_*i *_(for *A*_*i *_→ *A*_*i*+1_), gives the first example of limiting steps. Let the reaction rate constant *k*_*q *_be the smallest one. Then we expect the following behavior of the reaction chain in time scale ≳1/*k*_*q*_: all the components *A*_1_,...*A*_*q*-1 _transform fast into *A*_*q*_, and all the components *A*_*q*+1_,...*A*_*n*-1 _transform fast into *A*_*n*_, only two components, *A*_*q *_and *A*_*n *_are present (concentrations of other components are small), and the whole dynamics in this time scale can be represented by a single reaction *A*_*q *_→ *A*_*n *_with reaction rate constant *k*_*q*_. This picture becomes more exact when *k*_*q *_becomes smaller with respect to other constants.

The kinetic equation for the linear chain is(39)

The coefficient matrix *K *of these equations is very simple. It has nonzero elements only on the main diagonal, and one position below. The eigenvalues of *K *are -*k*_*i *_(*i *= 1,...*n *- 1) and 0. The left and right eigenvectors for 0 eigenvalue, *l*^0 ^and *r*^0^, are:(40)

all coordinates of *l*^0 ^are equal to 1, the only nonzero coordinate of *r*^0 ^is  and we represent vector-column *r*^0 ^in row.

The catalytic cycle is one of the most important substructures that we study in reaction networks. In the reduced form the catalytic cycle is a set of linear reactions:

Reduced form means that in reality some of these reaction are not monomolecular and include some other components (not from the list *A*_1_,... *A*_*n*_). But in the study of the isolated cycle dynamics, concentrations of these components are taken as constant and are included into kinetic constants of the cycle linear reactions.

For the constant of elementary reaction *A*_*i *_→ we use the simplified notation *k*_*i *_because the product of this elementary reaction is known, it is *A*_*i*+1 _for *i *<*n *and *A*_1 _for *i *= *n*. The elementary reaction rate is *w*_*i *_= *k*_*i*_*c*_*i*_, where *c*_*i *_is the concentration of *A*_*i*_. The kinetic equation is:(41)

where by definition *c*_0 _= *c*_*n*_, *k*_0 _= *k*_*n*_, and *w*_0 _= *w*_*n*_. In the stationary state (*ċ*_*i *_= 0), all the *w*_*i *_are equal: *w*_*i *_= *w*. This common rate *w *we call the cycle stationary rate, and(42)

where *b *= ∑_*i*_*c*_*i *_is the conserved quantity for reactions in constant volume. Let one of the constants, *k*_min_, be much smaller than others (let it be *k*_min _= *k*_*n*_):(43)

In this case, in linear approximation(44)

The simplest zero order approximation for the steady state gives(45)

This is trivial: all the concentration is collected at the starting point of the "narrow place", but may be useful as an origin point for various approximation procedures.

So, *the stationary rate of a cycle is determined by the smallest constant, k*_min_, if it is much smaller than the constants of all other reactions (43):(46)

In that case we say that **the cycle has a limiting step with constant ***k*_min_.

There is significant difference between the examples of limiting steps for the chain of reactions and for irreversible cycle. For the chain, the steady state does not depend on nonzero rate constants. It is just *c*_*n *_= *b*, *c*_1 _= *c*_2 _=... = *c*_*n*-1 _= 0. The smallest rate constant *k*_*q *_gives the smallest positive eigenvalue, the relaxation time is *τ *= 1/*k*_*q*_. The corresponding approximation of eigenmode (right eigenvector) *r*^1 ^has coordinates: . This exactly corresponds to the statement that the whole dynamics in the time scale ≳1/*k*_*q *_can be represented by a single reaction *A*_*q *_→ *A*_*n *_with reaction rate constant *k*_*q*_. The left eigenvector for eigenvalue *k*_*q *_has approximation *l*^1 ^with coordinates . This vector provides the almost exact *lumping *on time scale ≳1/*k*_*q*_. Let us introduce a new variable *c*_lump _= ∑_*i*_*l*_*i*_*c*_*i*_, i.e. *c*_lump _= *c*_1 _+ *c*_2 _+... + *c*_*q*_. For the time scale ≳1/*k*_*q*_we can write *c*_lump _+ *c*_*n *_≈ *b*, d*c*_lump_/d*t *≈ -*k*_*q*_*c*_lump_, d*c*_*n*_/d*t *≈ *k*_*q*_*c*_lump_.

In the example of a cycle, we approximate the steady state, that is, the right eigenvector *r*^0 ^for zero eigenvalue (the left eigenvector is known and corresponds to the main linear balance *b*:  ≡ 1). In the zero-order approximation, this eigenvector has coordinates 

If *k*_*n*_/*k*_*i *_is small for all *i *<*n*, then the kinetic behavior of the cycle is determined by a linear chain of *n *- 1 reactions *A*_1 _→ *A*_2 _→ ...*A*_*n*_, which we obtain after cutting the limiting step. The characteristic equation for an irreversible cycle, , tends to the characteristic equation for the linear chain, , when *k*_*n *_→ 0.

The characteristic equation for a cycle with limiting step (*k*_*n*_/*k*_*i *_<< 1) has one simple zero eigenvalue that corresponds to the conservation law ∑*c*_*i *_= *b *and *n *- 1 nonzero eigenvalues(47)

where *δ*_*i *_→ 0 when .

A cycle with limiting step (41) has real eigenspectrum and demonstrates monotonic relaxation without damped oscillations. Of course, without limitation such oscillations could exist, for example, when all *k*_*i *_≡ *k *> 0, (*i *= 1,...*n*).

**The relaxation time **of a stable linear system (41) is, by definition, *τ *= 1/min{*Re*(-*λ*_*i*_)} (*λ *≠ 0). For small *k*_*n*_, *τ *≈ 1/*k*_*τ*_, *k*_*τ *_= min{*k*_*i*_}, (*i *= 1,...*n *- 1). In other words, *for a cycle with limiting step, k_*τ *_is the second slowest rate constant*: *k*_min _<<*k*_*τ *_≤ ....

## Authors' contributions

AZ and AG have written the main body of the manuscript. NM, NN and AHB provided the critical review of miRNA mechanisms and contributed to writing the manuscript. AG and AZ developed the mathematical methodology for identifying dominant systems. AZ performed the analytical computations and numerical simulations. All authors have participated in discussing the results and model predictions. All authors read and approved the final manuscript.

## Supplementary Material

Additional file 1 **Analytical analysis of the case of very inefficient cap structure**. In this text we derive an asymptotical solution for the case when *k*_1 _is very small corresponding to the case of very inefficient translation initiation (for example, in the case of A-cap structure replacement experiment) Click here for file
