# Supplementary material for: Dynamical modeling of microRNA action on the protein translation process
Source: BMC Syst Biol. 2010 Feb 24;4:13. doi: 10.1186/1752-0509-4-13 (PMC2847993; doi:10.1186/1752-0509-4-13)
Supplement: Additional file 1 — Analytical analysis of the case of very inefficient cap structure. In this text we derive an asymptotical solution for the case when k1 is very small corresponding to the case of very inefficient translation initiation (for example, in the case of A-cap structure replacement experiment) [file 1752-0509-4-13-S1.PDF]

Supplementary Materials for the paper: Dynamical modeling of microRNA action on the protein translation process

Andrei Zinovyev [1,2,3,*], Nadezhda Morozova[4], Nora Nonne[4]
Emmanuel Barillot[1,2,3], Annick Harel-Bellan[4], Alexander Gorban[5]

[1] Institut Curie, S ervice Bioinformatique, Paris,France
[2] INSERM, U900, Paris, F-75248 France
[3] Ecole des Mines de Paris, ParisTech, Fontainebleau, France
[4] CNRS FRE 2944, Institut André Lwoff, Villejuif, France
[5] University of Leicester, UK
* Correspondent author e-mail: andrei.zinovyev@curie.fr

September 25, 2009

1 Analytical analysis of the case of very inefficient cap structure

We analyze the system of equations

$$\begin{cases} \dfrac{d\,[40S]}{dt} = -k_1[40S]\ [eIF4F] + k_4[80S] \\ \dfrac{d\,[eIF4F]}{dt} = -k_1[40S]\ [eIF4F] + k_2[mRNA:40S] \\ \dfrac{d\,[mRNA:40S]}{dt} = k_1[40S]\ [eIF4F] - k_2[mRNA:40S] \\ \dfrac{d\,[AUG]}{dt} = k_2[mRNA:40S] - k_3[AUG]\ [60S] \\ \dfrac{d\,[60S]}{dt} = -k_3[AUG]\ [60S] + k_4[80S] \\ \dfrac{d\,[80S]}{dt} = k_3[AUG]\ [60S] - k_4[80S] \\ Prsynth(t) = k_3[AUG]\ [60S] \end{cases} \quad (1)$$

with the following assumptions on the model parameters:

$$k_1 \ll k_4 \ll k_2 \ll k_3; [eIF4F]_0 \ll [40S]_0; [eIF4F]_0 < [60S]_0 < [40S]_0 \quad (2)$$

First of all, notice that, generally speaking, it is not eligible to compare the some parameters k_i: k_1 and k_3 has $\frac{1}{sec\_moles}$ dimensionality while k_2 and k_4 are $\frac{1}{sec}$. So instead of comparing k_1 and k_4, for example, one should rather compare, for example, $k_1[eIF4F]_0$ and k_4. To facilitate this task, we explicitly consider that $[eIF4F]_0$ is on the order of 10^0 in our model, $[60S]_0$ is at the order of 10^1 and $[40S]_0$ at 10^2.

1.1 Approximate steady state solution

From the conservation laws

$$[mRNA:40S]+[40S]+[AUG]+[80S]=[40S]_0, \tag{3}$$

$$[mRNA:40S]+[eIF4F]=[eIF4F]_0, \tag{4}$$

$$[60S]+[80S]=[60S]_0, \tag{5}$$

and the steady state condition

$$k_2\cdot[mRNA:40S]_s=k_3\cdot[AUG]_s\cdot[60S]_s=k_4\cdot[80S]_s=k_1\cdot[40S]_s\cdot[eIF4F]_s, \tag{6}$$

we can derive

$$\begin{aligned}
&[mRNA:40S]_s=\frac{k_4}{k_2}[60S]_0(1-x),[AUG]_s=\frac{k_4}{k_3}\left(\frac{1-x}{x}\right),\\
&[eIF4F]_s=[eIF4F]_0-\frac{k_4}{k_2}[60S]_0(1-x),\\
&[60S]_s=[60S]_0x,[80S]_s=[60S]_0(1-x),\\
&[40S]_s=[40S]_0-[60S]_0(1-x)(1+\frac{k_4}{k_2})-\frac{k_4}{k_3}\left(\frac{1-x}{x}\right)
\end{aligned} \tag{7}$$

where $x=\frac{[60S]_s}{[60S]_0}$ is the fraction of $60S$ in the free (unbound to mRNA) state.

Using (6) and (7) we obtain an equation on x:

$$\begin{aligned}
&x^3+\\
&+x^2\left(\alpha+(\delta-1)+(\beta-1)+\frac{[eIF4F]_0}{[60S]_0}\right)\frac{1}{1+k_4/k_2}+\\
&+x\left(-\alpha+(\delta-1)(\beta-1)+\frac{[eIF4F]_0}{[60S]_0}(-1-2\frac{k_4}{k_3[eIF4F]_0}+\frac{k_2}{k_3[60S]_0}+\frac{k_4}{k_2[eIF4F]_0})\right)\frac{1}{1+k_4/k_2}+\\
&+\gamma(1-\beta)=0,\\
&\alpha=\frac{k_2}{k_1[60S]_0},\beta=\frac{[eIF4F]_0k_2}{[60S]_0k_4},\gamma=\frac{k_4}{k_3[60S]_0},\delta=\frac{[40S]_0}{[60S]_0}
\end{aligned} \tag{8}$$

Having in mind $k_4 \ll k_2$ and assuming that $[eIF4F]_0/[60S]_0$ is sufficiently small, we simplify it to

$$x^3+x^2\left(\alpha+(\delta-1)+(\beta-1)\right)+x\left(-\alpha+(\delta-1)(\beta-1)\right)+\gamma(1-\beta)=0,. \tag{9}$$

From the inequalities on the parameters of the model, we have $\delta>1$, $\gamma\ll 1$, the constant term $\gamma(1-\beta)$ of the equation (9) should be much smaller than the other polynomial coefficients, and the equation (9) should have one solution close to zero and two others:

$$\begin{aligned}
&x_0\approx\frac{\gamma(\beta-1)}{-\alpha+(\beta-1)(\delta-1)}\\
&x_1=\frac{1}{2}\left(-(\alpha+\beta+\delta)+2+\sqrt{(\alpha+\beta+\delta)^2-4\beta\delta}\right)\\
&x_2=\frac{1}{2}\left(-(\alpha+\beta+\delta)+2-\sqrt{(\alpha+\beta+\delta)^2-4\beta\delta}\right)
\end{aligned} \tag{10}$$

.

If $k_1 \gg k_4/[eIF4F]_0$ then we have a situation already solved in the main body of the paper. Let us consider the opposite situation, when $k_1 \ll k_4/[eIF4F]_0$. In this case $\alpha \gg \beta + \delta$ and

$$\begin{aligned} x_0 &\approx \frac{\gamma(\beta - 1)}{-\alpha} = \frac{k_1 k_4}{k_3 k_2} - \frac{k_1 [eIF4F]_0}{k_3 [60S]_0} \\ x_1 &\approx 1 - \frac{\beta\delta}{\alpha} = 1 - \frac{k_1 [eIF4F]_0 [40S]_0}{k_4 [60S]_0} \\ x_2 &\approx -\alpha < 0 \end{aligned} \tag{11}$$

.

If $\alpha \gg \beta + \delta$ then the solution of the model can be approximated by the dominant system from Fig. 5 of the main body of the paper, Stage 1:

$$\begin{bmatrix} [eIF4F](t) \\ [mRNA:40S](t) \\ [AUG](t) \\ [80S] \end{bmatrix} = \frac{[eIF4F]_0}{\frac{1}{k_1'} + \frac{1}{k_2}} \left(\begin{bmatrix} 1/k_1' \\ 1/k_2 \\ 1/k_3' \\ 1/k_4 \end{bmatrix} + \frac{1}{k_2} \begin{bmatrix} 1 \\ -1 \\ 0 \\ \frac{k_2}{k_1' + k_2} \end{bmatrix} e^{-(k_1' + k_2)t} - \left(\frac{1}{k_4} + \frac{1}{k_1' + k_2}\right) \begin{bmatrix} 0 \\ 0 \\ 0 \\ 1 \end{bmatrix} e^{-k_4 t} \right) \tag{12}$$

and $[60S] = [60S]_0$, $[40S] = [40S]_0$. This solution is valid on the interval $[0; t']$, $t' = \frac{1}{k_1'+k_2} + \frac{[60S]_0}{10[eIF4F]_0}(\frac{1}{k_1'} + \frac{1}{k_2})$. Following the recipe from the main body of the paper, after this moment it can be prolonged with quasiequilibrium approximation:

$$\begin{aligned} &A = \frac{[eIF4F]_0}{k_4 \left(\frac{1}{k_1'} + \frac{1}{k_2}\right)} \\ &[80S](t) = \frac{[60S]_0}{10} + A\left(1 - e^{-k_4(t-t')}\right), [40S](t) = \frac{[40S]_0 - [80S](t)}{1 + \frac{k_1'}{k_2}[eIF4F]_0}, [60S](t) = [60S]_0 - [80S](t), \\ &[eIF4F](t) = \frac{k_2 \cdot [eIF4F]_0}{k_1 [40S](t)}, [mRNA:40S](t) = \frac{k_1 [40S](t) [eIF4F]_0}{k_2}, \\ &[AUG](t) = \frac{[eIF4F]_0}{\left(\frac{1}{k_1'} + \frac{1}{k_2}\right) k_3 ([60S]_0 - [80S](t))} \end{aligned} \quad . \tag{13}$$

Formulas (12-13) completely describes the dynamics of the system in the case $k_1 \ll k_4/[eIF4F]_0$. However, if $\alpha \approx \beta + \delta$ (i.e., when $k_1 \approx k_4/[eIF4F]_0$) then an other dominant system approximates the last stage of relaxation, when $[60S](t)$ becomes much smaller than $[AUG](t)$. Here the same quasi steady-state asymptotic as in the main body of the paper (Stage 2) is valid (see formula (42-44) of the main text). This completes the analysis of the system behaviour in the case when k_1 is relatively small with respect to other parameters. On the Fig. 1 one can see the comparison of these solutions with numerical simulations.

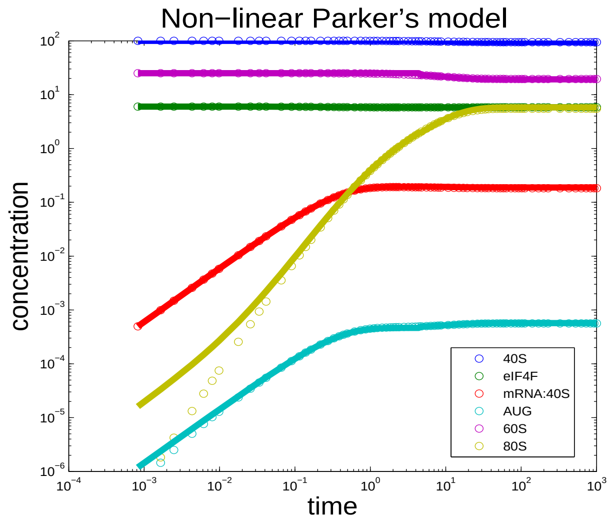

a)

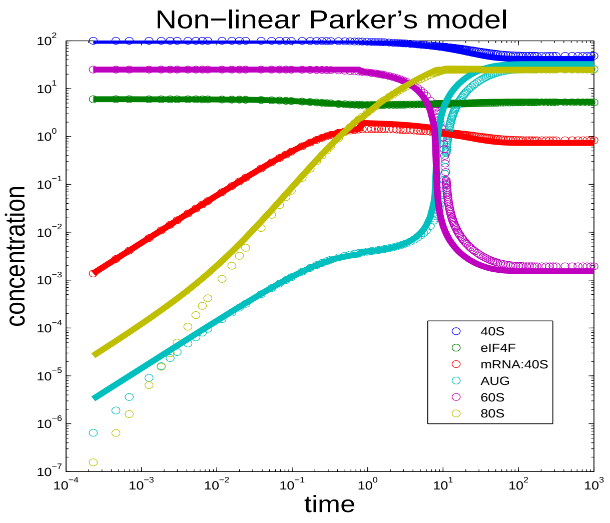

b)

Figure 1: a) Simulation of the non-linear protein translation model with parameters $k_1 = 0.001$, $k_2 = 3$, $k_3 = 50$, $k_4 = 0.1$, $[40S]_0 = 100$, $[60S]_0 = 25$, $[eIF4F]_0 = 6$. b) Same as a) but $k_1 = 0.01$. Circles represent the numerical simulation while solid lines gives the analytical solution.
